# Finger-Nail Signs

**Published:** 1861-04

**Authors:** 


Finger-Nail Signs.—In going round the wards of the Charite with M. Beau, a novice will be not a little puzzled at seeing him scrutinize closely the finger-nails of each newly admitted patient, telling him occasionally, after a few moments' examination of his cuticular appendage: "My friend, you had a bad illness so many months ago, a very severe illness that pulled you down a good deal; and then you had a relapse," and so on. This sort of inverted palmistry puzzled me sorely at first, and I confess that even the explanation, when given, left me very sceptical as to the infallibility of this retrospective fortune telling. Nevertheless, although I do not believe in Hume, the spirit-medium, any more than in Hahneman and his microdosic followers, I do believe in this sign of the past as indicated by the nails. If you look at the fingers of a man who had typhus fever three months ago, let us say, you will find on the nails, towards their centre (at the interval of time), a transverse furrow, deep and well-marked, coinciding with the moment when the check in their nutrition occurred—the depth of the depression being in proportion to the severity of the illness, its breadth with the duration; and the several consecutive relapses (if such occurred) being each notched on the ungual appendices as on so many tally-sticks. Few men know that they have the past history of their own cases so thoroughly at their fingers' end.—*London Lancet.—Savannah Med. Jour.*

